# Carbon monoxide poisoning with hippocampi lesions on MRI: cases report and literature review

**DOI:** 10.1186/s12883-024-03668-2

**Published:** 2024-05-11

**Authors:** Wenxia Li, Jun Meng, Jing Lei, Cheng Li, Wei Yue

**Affiliations:** 1 Clinical College of Neurology, Neurosurgery and Neurorehabilitation, Tianjin Medical University, Tianjin, 300070 China; 2 Tianjin Huanhu Hospital, Tianjin, 300350 China

**Keywords:** Hippocampi, Magnetic resonance imaging, Carbon monoxide, Poisoning, Case report

## Abstract

**Background:**

Carbon monoxide (CO) poisoning is now one of the leading causes of poisoning-related mortality worldwide. The central nervous system is the most vulnerable structure in acute CO poisoning. MRI is of great significance in the diagnosis and prognosis of CO toxic encephalopathy. The imaging features of CO poisoning are diverse. We report atypical hippocampal lesions observed on MRI in four patients after acute CO exposure.

**Case presentations:**

We report four patients who presented to the emergency department with loss of consciousness. The diagnosis of CO poisoning was confirmed on the basis of their detailed history, physical examination and laboratory tests. Brain MRI in all of these patients revealed abnormal signal intensity in hippocampi bilaterally. They all received hyperbaric oxygen therapy. The prognosis of all four patients was poor.

**Conclusion:**

Hippocampi, as a relatively rare lesion on MRI of CO poisoning, is of important significance both in the early and delayed stages of acute CO poisoning. In this article, we summarize the case reports of hippocampal lesions on MRI in patients with CO poisoning in recent years, in order to provide reference for the diagnosis and prognosis of CO poisoning.

## Background

Carbon monoxide (CO) is a colorless, tasteless, odorless gas that is imperceptible to human [1]. In several countries or regions, there is a high mortality rate due to CO poisoning. Of these, more patients die from unintentional CO poisoning than intentional reasons [2]. The central nervous system is the most vulnerable structure in acute CO poisoning due to its high energy demands [3]. MRI and related imaging modalities, the commonly used clinical imaging method, is important in assessing the severity of brain damage from CO poisoning and, to some extent, can predicts the prognosis of CO poisoning brain damage [4]. The imaging features of CO poisoning are diverse. It has been reported that the bilateral basal ganglia, especially the globus pallidus (GP), and the centrum semiovale are the most common sites of lesion in acute CO poisoning MRI, while cases involving the medial temporal lobe in the region of hippocampi are rare [4, 5]. We herein report four patients with CO poisoning who were studied by MRI during the acute phase and all were found to have lesions in the hippocampal region (Table 1).

**Table 1:** Clinical and laboratory data on four patients presenting with carbon monoxide poisoning

Patient No.	Gender/age (y)	Poisoning Cause	GCS	Carboxyhemoglobin (%)	BP (mmHg)	Time of 1st MRI (h after exposure)	Findings (location of lesions)	Restricted diffusion	Clinical findings	Past history of brain desease	Clinical effects	Treatment	Time between final CO exposure and first HBOT/ Number of HBOTs	Outcome/GCS at discharge
A	M, 65	Accidental	12	39	138/89	48	Hippocampi	Yes	Drowsy, intact pupillary light response, no responsive to verbal stimuli	No	Fluctuating illness including level of consciousness with dystonia and aphasia	Hyperbaric	3 h/5 separate days	Amnesia, aphasia, dystonia/15
B	M, 61	Accidental	10	19.2	120/77	24	Hippocampi, globus pallidus	Yes	Coma, unresponsive to verbal and pain stimuli, urinary/fecal incontinence	No	Coma, no examination data	Hyperbaric	4.2 h/8 separate days	Coma/8
C	M, 62	Accidental	8	7.8	124/85	72	Hippocampi	Yes	Coma, unresponsive to painful stimuli, poor pupillary light reflection	No	Amnesia, short-term memory loss	Hyperbaric	2.8 h/7 separate days	Amnesia/15
D	F, 24	Suicide	7	7	119/64	12	Medial temporal lobe, cerebral peduncle	Yes	Coma, unresponsive to verbal and pain stimuli, intact pupillary light response	Depression	Coma, no examination data	Hyperbaric	3.3 h/5 separate days	Lethargy with brief automatic eye opening/10

## Case presentations

**Fig. 1:**
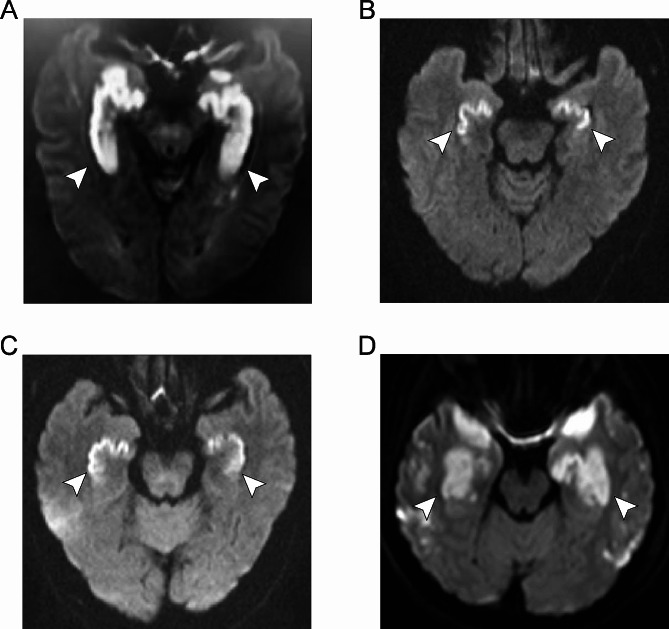
DWI obtained from four patients with acute carbon monoxide poisoning. (**A**) A 65-year-old man (patient A, COHb: 39%) examined at 48 h post-exposure, with demonstration of restricted diffusion (arrows) in the bilateral hippocampi. (**B**) A 61-year-old man (patient B, COHb: 19.2%) examined at 24 h post-exposure, with demonstration of restricted diffusion (arrows) in the bilateral hippocampi and globus pallidus. (**C**) A 62-year-old man (patient C, COHb: 7.8%) examined at 72 h post-exposure, with demonstration of restricted diffusion (arrows) in the bilateral hippocampi. (**D**) A 24-year-old woman (patient D, COHb: 7%) examined at 12 h post-exposure, with demonstration of restricted diffusion (arrows) in the bilateral medial temporal lobe and cerebral peduncle

Patient A:

A 65-year-old male was found unconscious and unresponsive to verbal stimuli in the house on 1/27/2018. The house he was in had a coal stove and the coals were burning incompletely. According to the investigation, he had suffered accidental CO poisoning as a result of a fault in the household’s heating. He was exposed in CO for approximately 15 h. There was no vomit beside him and he was not experiencing seizures. Then, he was transported to the hospital. In the emergency room, His vital signs were normal with the Glasgow coma score (GCS) 12. He was drowsy with an intact pupillary light response. He did not respond to verbal stimuli. His arterial carboxyl hemoglobin (COHb) was measured at 39%. Doctors treated him with hyperbaric oxygen therapy (HBOT) on 3 times. The patient became conscious 17 h after admission, responding to painful stimuli, and was able to move his limbs as instructed with GCS ranging from 12 to 15. Although he could understand words correctly, he still could not express himself. The patient received continuous HBOT on 5 separate days after admission. After 10 days of admission, his neurological function was largely restored. However, around the 20th day of hospitalization, the patient suddenly developed new neurological symptoms. He exhibited increased muscle tone, severely impaired cognitive function, memory loss, and mixed aphasia. Doctors considered him to have delayed neurological syndrome caused by CO poisoning. The MRI scan of his brain approximately 48 h after exposure to CO showed the bilateral hippocampi abnormalities (Fig. 1A).

Patient B:

A 61-year-old male was found unconscious and unresponsive to verbal stimuli inside his house on 12/29/2021. The house he was staying in had a coal stove for heating. And the carbon in the coal stove was burning incompletely. It is found that he was suffered from accidental CO poisoning after falling asleep. He was in the CO filled house for at least 10 h. He suffered from vomiting, urinary and fecal incontinence. His arterial COHb was 19.2%. After admission, the patient’s respiratory rate and heart rate were unstable, unresponsive to dizzand pain stimuli. And the patient was coma with GCS of 10. The patient received continuous HBOT during his hospitalization for 8 continuous days. During the subsequent hospitalization, the patient remained comatose with GCS ranging from 10 to 8. At the time of discharge, the patient’s consciousness was still in coma, unresponsive to verbal stimuli, and responsive to heavy pain stimuli. The MRI scan of the brain approximately 24 h after his exposure to CO showed the abnormalities in the bilateral hippocampi and basal ganglia (Fig. 1B).

Patient C:

A 62-year-old male was found unconscious and unresponsive to verbal stimuli in the house on 2/13/2023. He was beside a relatively large amount of vomit. He was unresponsive to painful stimuli. There were traces of burning charcoal in the house he was in and the person in the house with him was dead. This patient, along with the deceased in the same house, was accidentally poisoned with CO due to a house fire. His arterial COHb was 7.8%. The patient was comatose on admission with a GCS score of 8. His heart rate, respiratory rate and blood pressure were unstable. The patient’s pupillary light reflection was poor and his limbs did not respond to painful stimuli. The patient then received 2 HBO treatments. Twelve hours after admission, the patient’s mental state changed from coma to consciousness, but his cognitive function still did not recover, as exhibited by memory loss and unresponsiveness. The patient received continuous HBOT for 7 days. At the time of discharge, the patient’s consciousness shifted to conscious, with GCS ranging from 8 to 15, but he still had symptoms of memory loss. The MRI scan of the brain approximately 72 h after his exposure to CO showed abnormal signals in the bilateral hippocampi (Fig. 1C).

Patient D:

A 24-year-old female was found unconscious and unresponsive to verbal stimuli in the house on 2/16/2023. Her perioral area had white frothy secretions. Her family stated that she had been inside the house with a coal stove burning for warmth and that she had said she was dizzy. The patient was depressed. And this time she attempted suicide by burning coal. Her arterial COHb was 7%. On admission, the patient remained in a comatose state with a GCS score of 7, and her vital signs were unstable. The patient was unresponsive to verbal and pain stimuli, but the pupillary light reflection was present. During her hospitalization, the patient received continuous hyperbaric oxygen therapy for 5 times. At the time of discharge, the consciousness of the patient changed from coma to lethargy with brief spontaneous eye opening. And GCS was ranging from 7 to 10. The MRI scan of the brain approximately 12 h after her exposure to CO showed abnormal signals in the medial temporal lobe and cerebral peduncle (Fig. 1D).

## Literature review and discussion

The pathophysiologic mechanism of CO poisoning involves the binding of CO to hemoglobin to form COHb [6]. CO shows a 250-fold higher affinity for hemoglobin than oxygen and competitively binds to it to form COHb. COHb has no ability to carry oxygen and is not easily dissociated, which also shifts the hemoglobin oxygenation curve to the left. In this case, blood oxygen is not easily released to the tissues, resulting in cellular hypoxia [7, 8]. At the same time, CO affects mitochondrial metabolism, which can aggravate tissue hypoxia [9]. Furthermore, studies have shown that hypotension and cardiac dysfunction induced by CO also can lead to circulatory hypoxia in the body [10, 11]. The pathological changes in the brain tissue of patients with CO poisoning are similar to those of hypoxic encephalopathy, like cerebral edema and varying degrees of necrosis. Cerebral edema and ischemia can be followed by cerebral circulatory disorders, causing ischemic cerebral necrosis and further aggravating cerebral hypoxia [4].

CO poisoning causes a wide variety of symptoms that exhibit an unspecific character. Patients often present with tachycardia, headache, vomiting, fainting, and seizures [12]. Studies have shown that approximately 20% of patients with CO poisoning experience a progression from acute to chronic symptoms, and approximately 10% develop delayed neurological syndrome [13]. Some scholars have categorized the degree of poisoning as mild, moderate, or severe based on COHb concentration [14]. Hence, concentration of COHb seems to be somewhat proportional to the severity of clinical symptoms, as seen in the four patients we reported. However, it has been found that concentration of COHb is closely related to that of CO in the air at the time of intoxication and the duration of exposure, whereas the degree of intoxication is not only related to the concentration of COHb, but also to the the clinical manifestations, especially the individual’s tolerance to hypoxia [15]. Therefore, COHb concentration cannot be used to determine the severity of the patient’s symptoms and prognosis.

MRI, one of the most commonly used imaging methods, is of great significance in the diagnosis and prognosis of CO toxic encephalopathy. MRI can provide an objective assessment of brain damage [16]. Some researchers defined the time between CO exposure and MRI as the hyperacute phase within 24 h, the acute phase between 24 h and 7 days, the subacute phase between 8 and 21 days, and the chronic phase over 22 days [4]. The early stage of acute CO toxic encephalopathy on MRI mainly involves the cerebral white matte (CWM) and basal ganglia (especially the GP) [17]. Current conventional MRI studies have focused on some typical manifestations in the chronic phase, i.e., typical findings of bilateral high signal in basal ganglia and CWM on T2WI-weighted images [18]. Many studies have shown that magnetic resonance DWI can help to assess ischemic-hypoxic brain damage in both the hyperacute and acute phases of CO poisoning. DWI can characterize cell toxic edema in damaged CWM more sensitively and earlier than conventional MRI [15]. Moon et al. [19] found that DWI could reflect cytotoxic edema after CWM injury more sensitively than conventional MRI and may contribute to the prediction of long-term neurologic outcomes after discharge from the hospital.

With regard to damage to gray matter structures other than the GP during the acute phase of CO poisoning, few reports have described hippocampal lesions, and most of the cases were combined with other areas of abnormality. Of the 19 patients with acute CO poisoning reported by Donnell et al. [20], four patients exhibited abnormal signals in medial temporal lobe in the region of hippocampi, most of which were bilateral and combined with abnormalities in other locations. Kim et al. [15] reported the DWI imaging characteristics of 7 patients with acute phase of CO poisoning, of whom 2 had hippocampal lesions that showed limited diffusion of the lesion site on the DWI/ADC maps and their symptoms manifested as lethargy or coma. Henke et al. [21] reported a patient with CO poisoning who showed undefined high signal in the hippocampal lesions bilaterally on T2-weighted images 5 days after poisoning. His symptoms presented with severe amnesia and disorientation. There also have been previous reports describing damage to hippocampi in the chronic phase in patients with CO poisoning. Bastin et al. [22] performed brain MRI in a patient with CO poisoning 18 years after the event. The results showed that the hippocampal volume of this patient was reduced by more than 50% compared to a normal healthy population. The patient’s clinical presentation was characterized as severely impaired recall. Tamura et al. [23] examined MRI of a patient with acute CO poisoning 1 year after the event. The rate of hippocampal volume reduction in the first year after CO poisoning was approximately 4% compared to her previous MRI. Furthermore, in a report on hippocampal lesions revealed that acute phase hippocampal lesions may portend a very poor prognosis [20]. In all four patients we reported, the first MRI was performed within 72 h of the event, and DWI imaging showed that all patients presented with bilateral hippocampal lesions with or without abnormalities in other areas. The four patients we reported had clinical signs of impaired consciousness in the acute phase, two patients developed cognitive dysfunction with severe impairment of short-time memory in the acute phase, and two patients suffered from a persistent coma. All four patients were treated with hyperbaric oxygen, reduction of cerebral edema, and improvement of coronary flow, but the prognosis was not good, and one of them developed delayed neurological syndrome, which is similar to the report of Donnell et al [20].

Most of these patients with hippocampal lesions after CO poisoning reported above developed cognitive dysfunction during the recovery period, and most had a poor prognosis. The hippocampus is an important region for memory, learning, and emotional activities and has a high metabolic rate. Therefore, the hippocampus is very vulnerable to ischemia and hypoxia. In the animal model of acute CO poisoning, the hippocampal neurons of CO-poisoned rats were obviously damaged and hippocampal neurogenesis were significantly inhibited, which is consistent with the imaging performance of the cases reported in this study. Therefore, we conducted a systematic review of MRI of CO poisoning showing abnormal lesions in hippocampi (Table 2). The results found that, firstly, isolated bilateral hippocampal lesions are rare in the acute phase of poisoning, and most of them were reported as unilateral or bilateral hippocampal abnormalities combined with lesions in other parts of the brain, whereas the two patients reported in the present study had isolated bilateral hippocampal lesions; secondly, most of the patients with hippocampal lesions had cognitive dysfunctions in their clinical presentation, and two of the patients reported in the present study developed cognitive dysfunctions; finally, the hippocampal lesions in acute phase may portend a poor prognosis; in the present case, two patients had remaining cognitive dysfunction, and two patient was in lethargy or coma state.

**Table 2:** Case studies with MRI of carbon monoxide poisoning showing abnormal lesions in hippocampi

Study	Region	Gender, age (y)	GCS	Carboxyhemoglobin (%)	BP (mmHg)	Time of 1st MRI (after exposure)	Findings (location of lesions)	Restricted diffusion	Clinical effects	Treatment	Outcome
Henke et al. (1999)	Switzerland	M,28	5	54	156/88	120 h	GP, hippocampus	NA	Lethargy, psychomotor retardation, unorientation	Hyperbaric	Amnesic
		M,26	NA	34.8	NA	Less than 4 days	GP, hippocampus, amygdala, CBLM, CWM	Yes	Mental status change (semi-coma)	Oxygen	NA
Bastin et al. (2014)	Belgium	M,65	NA	10.8	NA	18 days	Both hemispheres	NA	Confused, cognitive difficulties	NA	Memory disorders
Donnell et al. (2000)	England	M,47	6	27.8	109/78	22 h	GP, BG, MTL	NA	NA	NA	Short-term memory loss
		M,51	4	64	150/100	19 h	GP, MTL, WM, CBLM	NA	NA	NA	Died following transfer
		F,21	7	7	130/80	37 h	GP, BG, MTL, CBLM	NA	NA	NA	Died day 5
		M,33	3	15.3	NA	33 h	GP, MTL, WM, CTX	NA	NA	NA	Aphasia, ataxia
Kim et al. (2017)	South Korea	M,31	NA	8.3	NA	Less than 3 day	GP, hippocampus, CBLM, TWM	Yes	Mental status change (drowsy)	Hyperbaric	NA
		M,26	NA	34.8	NA	Less than 4 day	GP, hippocampus, amygdala, CBLM, CWM,	Yes	Mental status change (semi-coma)	Oxygen	NA
Tamura et al. (2020)	Japan	F,22	NA	24.8	NA	1 year	Hippocampus	No	Headaches, dizziness, dyspnea, and drowsiness	Hyperbaric	Memory disorders

NA: not availabie; GP: globus pallidus; CWM: cerebral white matte; BG: basal ganglia; MTL: medial temporal lobe; TWM: temporal white matter; CBLM: cerebellum

## Conclusion

Our cases illustrated that the hippocampi, as an atypical presentation, can be seen on MRI in a few patients with CO poisoning. Hippocampal lesions indicated by MRI are extremely significant in acute and subacute phases in patients with acute CO toxic brain injury. MRI is able to detect hippocampal lesions in acute injuries with sudden onset of neurologic deficits, and may also be able to predict, to some extent, brain injury in patients in the mid- to long-term, suggesting a prognosis for the patient. In this article, we summarize the case reports of hippocampal lesions on MRI in patients with CO poisoning in recent years, in order to provide reference for the diagnosis and prognosis of CO poisoning.

## Data Availability

Data sharing is not applicable to this article as no datasets were generated or analysed during the current study.

## Abbreviations

BG: basal ganglia
CO: carbon monoxide
COHb: carboxyl hemoglobin
CBLM: cerebellum
CWM: cerebral white matte
GP: globus pallidus
GCS: Glasgow coma score
HBOT: hyperbaric oxygen therapy
MTL: medial temporal lobe
TWM: temporal white matter
